# Anti-type II collagen immune complex-induced granulocyte reactivity is associated with joint erosions in RA patients with anti-collagen antibodies

**DOI:** 10.1186/s13075-015-0523-7

**Published:** 2015-01-19

**Authors:** Vivek Anand Manivel, Azita Sohrabian, Marius C Wick, Mohammed Mullazehi, Lena Douhan Håkansson, Johan Rönnelid

**Affiliations:** Department of Immunology, Genetics and Pathology, Uppsala University, Uppsala, Sweden; Department of Radiology, Karolinska University Hospital, Stockholm, Sweden; Department of Medical Sciences, Uppsala University, Uppsala, Sweden; Unit of Rheumatology, Department of Medicine, Karolinska Institutet, Stockholm, Sweden

## Abstract

**Introduction:**

Rheumatoid arthritis (RA) patients with autoantibodies against collagen type II (CII) are characterized by acute RA onset with elevated inflammatory measures and early joint erosions as well as increased production of tumor necrosis factor-α (ΤΝF-α) by peripheral blood mononuclear cells (PBMC) stimulated by anti-CII immune complexes (IC) *in vitro*. Polymorphonuclear granulocytes (PMN) are abundant in RA synovial fluids, where they might interact directly with anti-CII IC in the articular cartilage, but no studies have investigated PMN responses towards anti-CII IC. The aim was to investigate whether PMN react towards anti-CII IC, and to what extent such reactivity might relate to the clinical acute onset RA phenotype associated with elevated levels of anti-CII.

**Methods:**

PMN and PBMC isolated from healthy donors were stimulated with IC made with a set of 72 baseline patient sera (24 anti-CII positive, 48 anti-CII negative) chosen from a clinically well-characterized RA cohort with two-year radiological follow-up with Larsen scoring. PMN expression of cluster of differentiation (CD)11b, CD66b, CD16 and CD32 was measured by flow cytometry, whereas PMN production of myeloperoxidase (MPO) and interleukin (IL)-17, and PBMC production of ΤΝF-α was measured with enzyme linked immunosorbent assay.

**Results:**

PMN expression of CD11b, CD66b and MPO, and PBMC production of ΤΝF-α were upregulated whereas PMN expression of CD16 and CD32 were downregulated by anti-CII IC. CD16, CD66b, and MPO production correlated to serum anti-CII levels (Spearman’s ρ = 0.315, 0.675 and 0.253, respectively). CD16 was associated with early joint erosions (*P* = 0.024, 0.034, 0.046 at baseline, one and two years) and CD66b was associated with changes in joint erosions (*P* = 0.017 and 0.016, at one and two years compared to baseline, respectively). CD66b was associated with baseline C-reactive protein and PBMC production of ΤΝF-α was associated with baseline erythrocyte sedimentation rate, in accordance with our earlier findings. No clinical associations were observed for MPO or IL-17.

**Conclusion:**

PMN responses against anti-CII IC are more closely associated with early joint erosions than are PBMC cytokine responses. PMN reactivity against anti-CII IC may contribute to joint destruction in newly diagnosed RA patients with high levels of anti-CII.

## Introduction

Rheumatoid arthritis (RA) is a chronic inflammatory disease characterized by joint pain and destruction. Collagen type II (CII) is a major component of joint cartilage. We have described a distinct acute-onset RA phenotype characterized by high levels of collagen type II autoantibodies (anti-CII) in serum and increased C-reactive protein (CRP) levels and erythrocyte sedimentation rate (ESR) at the time of diagnosis [1]. Shortly after diagnosis RA patients’ anti-CII levels drop, as do levels of CRP and ESR. Anti-CII-positive patients also have more joint destructions and a shorter time from symptom onset to clinical RA diagnosis, further arguing for a more acute onset among these patients [1,2]. We have previously described an *in vitro* model showing that anti-CII-containing immune complexes (ICs) induce tumor necrosis factor alpha (ΤΝFα), interleukin (IL)-1β and IL-8 from peripheral blood monocytes via Fc gamma receptor IIa [3]. We hypothesized that these IC-induced cytokines drive an early acute phase response and joint destruction, and in agreement with this hypothesized that serum anti-CII levels, *in vitro* ΤΝFα and IL-1β production, ESR, and CRP show temporal associations in serially followed patients with high baseline anti-CII levels [1].

The T-cell-dependent and B-cell-dependent collagen-induced arthritis mouse model has substantiated the possible importance of collagen immunity in RA [4]. The strictly humoral collagen antibody-induced arthritis (CAIA) mouse model shows that anti-CII can initiate arthritis [5,6]. CAIA has been induced in mice lacking an adaptive immune system, showing a direct role of anti-CII in arthritis development [7]. Our hypothesis is that CAIA is a mouse model for the acute-onset anti-CII-dependent RA phenotype.

Polymorphonuclear granulocytes (PMN) are found in large numbers in synovial fluid in active RA. These PMN are reactive and affect cytokine-signaling pathways [8-11]. Although PMN normally are short lived and easily undergo apoptosis, in the synovial environment they are influenced by cytokines and other factors increasing survival [12,13]. Studies in juvenile idiopathic arthritis implicate PMN in arthritis development [14-16]. During inflammation, PMN respond by producing reactive oxidative species and cytokines including IL-17 [17], releasing myeloperoxidase (MPO) and other granule proteins, and by regulating cell surface markers. PMN express Fc gamma receptors including CD16 that bind ICs, generating intracellular signals through immunoreceptor-based tyrosine motifs [18-22]. Other PMN surface molecules such as CD11b and CD66b are effective in adhesion to endothelium during inflammatory processes. CD66b is upregulated by phorbol mysistate acetate or by *N*-formyl-methionyl-leucyl-phenylalanine *in vitro*, and is also upregulated on RA PMN both in the circulation [23] as well as in synovial fluid [24]. PMN communicate with the adaptive immune system through chemokines such as CXCL8 [25] and IL-17 [17,26,27]. IL-17 has been known to be involved in the pathogenesis of autoimmune diseases [28], and blockade of IL-17 modulated autoimmunity [29], including arthritis [30].

PMN are also found in the pannus tissue, thus in close contact with cartilage where anti-CII-containing ICs can form and be involved in cartilage damage and joint destruction [31-33]. We therefore wanted to study PMN functional reactivity towards anti-CII ICs. Experiments were designed to study this hypothesized mechanism initiated by the interaction of PMN with anti-CII IC, and to address the role of PMN in the distinct anti-CII-dependent acute-onset RA phenotype.

## Methods

### Patients and cell donors

RA patients were chosen from a cohort of 274 RA patients included from 1995 to 2000 at the Department of Rheumatology, Karolinska University Hospital as described previously [1]. All patients fulfilled the 1987 American College of Rheumatology classification criteria [34]. From this group a smaller set of 72 samples manageable for functional studies were chosen. This group includes all 24 anti-CII-positive patients in the full cohort, the 24 patients with the lowest anti-CII (negative) and rheumatoid factor (RF)-negative, and the 24 patients with the lowest anti-CII (negative) and RF-positive (Table 1). The latter subdivision was made to rule out any effects of RF on *in vitro* functional responses to ICs, due to nonspecific IgG binding to the wells that we have found mostly in anti-CII-negative samples [3]. The baseline clinical presentation of the full RA cohorts has been published before [1], and the presently investigated 72 patients are detailed in Table 1. Identical X-ray images of hands and feet were obtained at baseline, 1 year, and 2 years, and were quantified in a blinded manner by an experienced investigator (MCW) using the Larsen erosion score as described earlier [2]. Changes in Larsen score were calculated by subtracting either the baseline or the 1-year Larsen score from the 2-year or 1-year scores. Heparinized blood was collected from healthy donors and laboratory staff at Uppsala University Hospital. The regional ethical boards at Uppsala University and Karolinska University Hospital approved the research, and all patients and cell donors gave informed consent.

**Table 1:** **Baseline characteristics of the 72 included rheumatoid arthritis patients**

	**All RA patients (** ***n*** **= 72)**	**Anti-CII-negative RA patients (** ***n*** **= 48)**	**Anti-CII-positive (>29 AU/ml) RA patients (** ***n*** **= 24)**	***P*** **(between anti-CII-negative and all anti-CII-positive patients)**	**Anti-CII highly positive (>470 AU/ml) RA patients (** ***n*** **= 9)**	***P*** **(between anti-CII-negative and highly anti-CII-positive patients)**
Mean age at onset (years)	59.6 (14.4)	59.7 (15.0)	59.4 (30.5)	NS	63.3 (13.7)	NS
% females	76 (55/72)	79.2 (38/48)	70.8 (17/24)	NS	66.7 (6/9)	NS
Mean disease duration at inclusion (months)	5.5 (2.6)	6.1 (2.8)	4.2 (1.6)	**0.005**	4.4 (1.4)	NS (0.08)
RF-positive (%; number positive/total number)	51 (36/71)	50 (24/48)	52 (12/23)	NS	44 (4/9)	NS
Anti-CCP2-positive, (%; number positive/total number)	47 34/72	48 (23/48)	37.5 (9/24)	NS	33.3 (3/9)	NS
CRP (mg/l)	31 (40)	20 (18)	53 (60)	**0.020**	46 (34)	**0.007**
ESR (mm/hour)	28 (22)	24 (18)	36 (27)	NS	41 (27)	**0.039**
Physician’s assessment of disease activity (0 to 4)	2.1 (0.8)	2.0 (0.8)	2.3 (0.6)	NS	2.4 (0.5)	NS
Number of swollen joints	10 (5)	8 (5)	12 (6)	**0.022**	10 (5)	NS
Number of tender joints	9 (5)	9 (5)	8 (6)	NS	8 (3)	NS
DAS28	5.1 (1.1)	5.0 (1.0)	5.2 (1.3)	NS	5.5 (0.9)	NS
Global VAS	43 (27)	45 (27)	39 (28)	NS	46 (29)	NS
Pain VAS	45 (23)	49 (23)	38 (21)	NS	37 (21)	NS
HAQ	0.99 (0.59)	0.97 (0.58)	1.02 (0.61)	NS	1.33 (0.55)	NS
Baseline Larsen score	9.71 (10.42)	9.36 (10.69)	10.37 (10.05)	NS	12.28 (6.52)	NS (0.12)
% of patients starting DMARD therapy at baseline	92 (66/72)	96 (46/48)	83 (20/24)	NS	89 (8/9)	NS

Data presented as means values (standard deviation) and as percentages (ratios). Differences between anti-CII-negative and anti-CII-positive patients are analyzed using Mann–Whitney’s *U* test, whereas differences between proportions are analyzed using the chi-square test or Fisher’s exact test when appropriate. Data for anti-CCP and RF have been published earlier [35,36]. Data for RF at the time of diagnosis are missing for one patient. Significant values are in bold. We have deliberately expressed mean values to allow comparison with previously published data on the full RA cohort ([3]; *n* = 274). anti-CCP2, cyclic citrullinated peptide version 2 antibodies; anti-CII, collagen type II autoantibodies; AU, arbitrary units; CRP, C-reactive protein; DAS28, Disease Activity Score in 28 joints; DMARD, disease-modifying antirheumatic drug; ESR, erythrocyte sedimentation rate; HAQ, Health Assessment Questionnaire; NS, nonsignificant; RA, rheumatoid arthritis; RF, rheumatoid factor; VAS, visual analog scale.

### Preparation of immune complex

Cytokine stimulation by solid phase IC was performed as described previously [3]. Native human CII (Chondrex, Redmond, WA, USA) was diluted to 10 μg/ml and 50 μl were added to Maxisorb enzyme-linked immunosorbent assay (ELISA) wells (Nunc, Roskilde, Denmark). After overnight incubation at +4°C, plates were blocked with 1% human serum albumin for 1 hour. Thereafter 50 μl of 10% patient sera diluted in phosphate-buffered saline were added for 2 hours at room temperature for stimulation of peripheral blood mononuclear cells (PBMC), whereas undiluted sera were used to prepare surface-bound IC for stimulation of PMN. After washing, 300 μl responder PBMC (10^6^/ml) and PMN (10^6^/ml) respectively were added to the plates that were left to incubate for 20 hours in a cell incubator, and then cells were harvested for flow cytometry and supernatants for measurement of soluble substances. To prepare ICs, new serum aliquots never thawed or thawed/frozen only once were obtained from the Karolinska biobank. A serum from the same RA patient that had been used to establish the standard curve in the anti-CII ELISA was used in making anti-CII IC used in the initial experiments defining PMN responses.

### Cell purification

In initial experiments, PMN were purified from sodium heparinized blood (Greiner bio-one GmbH, Kremsmünster, Austria) with Ficoll (GE Healthcare, Uppsala, Sweden) after osmotic lysis of the red blood cells. In later experiments, PMN were isolated by Percoll (GE Healthcare) gradients with 72% Percoll at the bottom and 63% Percoll at the top. In this method the PMN were obtained between the two Percoll gradients. PBMC were initially isolated by Ficoll as done in our earlier studies [3] and in later experiments from the upper part of the Percoll gradient described above. The cell count and viability was checked with Türk’s solution and trypan blue solution respectively in a Bürker chamber. The purity and viability obtained from the two cell separation protocols were comparable. Both the PMN and PBMC had purity above 96% and viability above 94% (PBMC) and 91% (PMN).

### Human collagen type II IgG ELISA

Data on anti-CII antibody levels were obtained from our previous studies [1]. Patient samples above the 95th percentile among controls (29 arbitrary units (AU)/ml) were regarded as positive [1]. In some experiments, known concentrations of polyclonal IgG where the source was a pharmaceutical preparation intended for intravenous immunoglobulin therapy (Privigen; CSL Behring, Stockholm, Sweden) were serially diluted, coated on ELISA plates and used as a standard curve to translate the concentration of anti-CII from arbitrary units per milliliter to micrograms of anti-CII IgG per milliliter. Privigen-coated ELISA wells were also used to stimulate PBMC and PMN in parallel to anti-CII IC stimulation, to allow comparison of the functional effect of anti-CII IgG with mixed human IgG.

### Cell stimulation

ELISA plates were coated with native human CII (10 μg/ml) (ELISA grade; Chondrex Inc.) at 4°C overnight. The anti-CII IC was prepared by adding RA sera to the CII-coated ELISA plates during 2 hours of incubation on a shaker at room temperature.

In a first set of experiments, aimed at defining appropriate measures for a clinical study, PMN isolated from eight healthy donors were subjected to IC stimulation with a serum with high concentration of anti-CII in IC (14740 AU/ml or 8 μg/ml) or with an anti-CII-negative healthy control serum. In the second experiment, where functional responses were related to clinical outcome, PMN and PBMC obtained from a single donor was stimulated with anti-CII IC prepared with sera from 72 RA patients.

PBMC were stimulated with anti-CII IC prepared with 50 μl sera diluted 1:10 in each ELISA well as in previous studies [3]. Initial experiments showed that PMN needed higher antibody IgG concentrations for functional responses, and therefore undiluted sera were used in otherwise identical ICs to stimulate PMN. Cells for flow cytometry and supernatants for cytokine and MPO measurements were harvested after 18 hours in a cell incubator, a time determined as optimal for the investigated the CD markers.

### Flow cytometry analysis

Flow cytometry analysis was carried out with fluorescein isothiocyanate-conjugated IgG_1_ antibodies against CD11b (Bear1; Beckman Coulter, Marseille, France), CD66b (80H3; Beckman Coulter), CD16 (DJ130c; DAKO, Glostrup, Denmark), and phycoerythrin-conjugated antibodies against CD32 (2E1; Beckman Coulter). After addition to cells, tubes were kept for 30 minutes at 4°C before washing and fixing with 1% paraformaldehyde. Color compensation of phycoerythrin and fluorescein isothiocyanate was done with anti-mouse immunoglobulin, κ/negative control compensation particle set (BD Biosciences. Nonspecific binding was checked with IgG_1_ isotype controls (Beckman Coulter). Analyses were performed with a BD FACSCanto II flow cytometer and FACSDIVA software (BD Biosciences).

### Cytokine and myeloperoxidase measurements

Cytokine measurements were carried out with the ELISA protocol used in previous studies [1,3]. All antibodies and cytokine standards were from R&D Biosciences (Abingdon, UK). For ΤΝFα measurement, the capture antibody was mouse monoclonal MAB610, (2.0 μg/ml) and the detection antibody was biotinylated polyclonal goat antibody BAF210 (0.1 μg/ml). For IL-17, the Duo-set kit was employed, using recommended antibody concentrations. MPO was analyzed using an ELISA kit from Diagnostic AB (Uppsala, Sweden) and performed according to the instructions from the manufacturer.

### Statistical analysis

Because we have shown previously that anti-CII levels are non-normally distributed [2], we uniformly used nonparametric statistics. Groups were compared using the Mann–Whitney *U* test, paired investigations were done with the Wilcoxon signed-rank test, and correlations were calculated with Spearman’s rank correlation test. When changes in responses against anti-CII ICs were calculated, the responses in anti-CII-coated wells treated with a normal human (anti-CII-negative) serum were subtracted from the responses against anti-CII IC. There are no common rules for how to evaluate association between functional responses (CD16, CD66b, ΤΝFα, MPO) and clinical measures presented in Table 2, so these responses were initially evaluated with different cutoff values corresponding to the 50th to 95th percentiles of the maximal responses. The 85th percentile was uniformly found to be the most discriminatory cutoff point for all responses, numerically delimiting a fraction of patients corresponding to those with very high anti-CII levels. All calculations were performed with Graphpad prism 6 and JMP 9.0 software. *P* <0.05 was considered significant. No correction for mass significance was done.

**Table 2:** **Association between, on one hand,** ***in vitro*** **granulocyte responses, PBMC responses and baseline antibody levels and, on the other, baseline inflammatory markers, Larsen score and changes in Larsen score during the first 2 years after RA diagnosis**

	**Baseline CRP**	**Baseline ESR**	**Baseline Larsen score**	**1-year Larsen score**	**2-year Larsen score**	**∆ Larsen score 1 – 0 years**	**∆ Larsen score 2 – 0 years**	**∆ Larsen score 2 – 1 years**
CD16 (PMN)	35/16.5 (0.08)	35.5/19.5 (0.26)	**14.4/5.5 (0.024)**	**22.5/11.0 (0.034)**	**24.0/31.1 (0.046)**	7.3/3.0 (0.08)	9.0/6.3 (0.88)	3.0/2.3 (0.27)
CD66b (PMN)	**49/14 (0.004)**	33/19 (0.063)	13.5/6.0 (0.15)	22.0/11.0 (0.071)	24.0/13.2 (0.059)	**7.5/2.6 (0.017)**	**10.4/5.2 (0.016)**	**5.1/2.3 (0.012)**
MPO (PMN)	14/18 (0.6)	19/21 (0.61)	5.4/7.0 (0.69)	17.0/11.0 (0.99)	17.7/13.2 (0.97)	6.9/2.8 (0.27)	9.6/5.3 (0.41)	2.5/2.3 (0.37)
ΤΝFα (PBMC)	28/17 (0.18)	**43/19 (0.049)**	9.3/6.3 (0.57)	11.6/11.8 (0.75)	15.3/13.2 (0.52)	5.6/3.0 (0.50)	9.4/5.3 (0.43)	2.9/2.3 (0.48)
Anti-CII	**36.5/14 (0.012)**	29/19 (0.10)	11.5/6.3 (0.75)	16.0/11.0 (0.44)	15.0/13.5 (0.44)	4.3/3.6 (0.21)	8.1/5.3 (0.22)	2.6/2.3 (0.29)
Anti-CCP	15/18.5 (0.20)	23/20 (0.57)	6.8/7.0 (0.76)	13.0/10.5 (0.76)	15.8/12.0 (0.42)	4.6/2.5 (0.41)	8.3/5.3 (0.12)	**3.0/1.5 (0.024)**
RF	15/18 (0.65)	23/20 (0.66)	6.0/7.9 (0.17)	11.0/12.0 (0.52)	14.0/13.5 (0.64)	4.4/3.6 (0.71)	6.3/7.0 (0.44)	**3.0/1.8 (0.049)**

Data presented as median level for patients with significant change/not significant change in the parameter shown in the left column. Statistics were performed with the Mann–Whitney *U* test. *P* values are shown within parentheses; significant values are in bold. anti-CII, collagen type II autoantibodies; anti-CCP, cyclic citrullinated peptide antibodies; CRP, C-reactive protein; ESR, erythrocyte sedimentation rate; MPO, myeloperoxidase; PBMC, peripheral blood mononuclear cells; PMN, polymorphonuclear granulocytes; RA, rheumatoid arthritis; RF, rheumatoid factor; TNFα, tumor necrosis factor alpha.

## Results

### Polymorphonuclear granulocytes respond to anti-CII-containing surface-bound immune complex by changes in expression of CD markers

**Figure 1:**
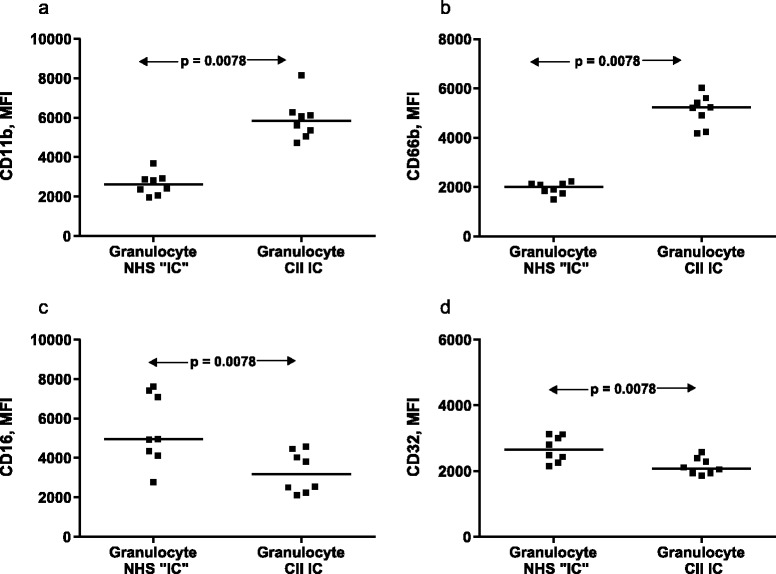
**Granulocyte expression of CD11b, CD66b, CD16, and CD32.** Granulocyte expression of **(a)** CD11b, **(b)** CD66b, **(c)** CD16, and **(d)** CD32 after 18 hours of stimulation with surface-bound collagen type II autoantibody (anti-CII) immune complex (IC) prepared with a high-level anti-CII serum or with normal human serum. Eight healthy donors provided the cells. Horizontal bars, median values. Statistical comparisons were made with the paired Wilcoxon signed-rank test. CII, collagen type II; MFI, mean fluorescence intensity; NHS, normal human serum.

PMN cell surface CD11b and CD66b were upregulated and CD16 and CD32 were downregulated by anti-CII IC stimulation. When eight PMN donors were investigated in parallel, the median change in mean fluorescence intensity was +141% for CD11b, +165% for CD66b, –41% for CD16 and –17% for CD32 (Figure 1). All eight PMN preparations displayed the same pattern of response for each CD marker. Directly coated IgG induced changes similar to those of anti-CII IC (data not shown). From these experiments and initial titrations performed with various concentrations of anti-CII IC, CD66b and CD16 were proven to be the most sensitive markers of PMN reactivity and were henceforth used in our clinical cohort study.

### Comparison between stimulatory effects of surface bound anti-CII immune complex and directly coated IgG

**Figure 2:**
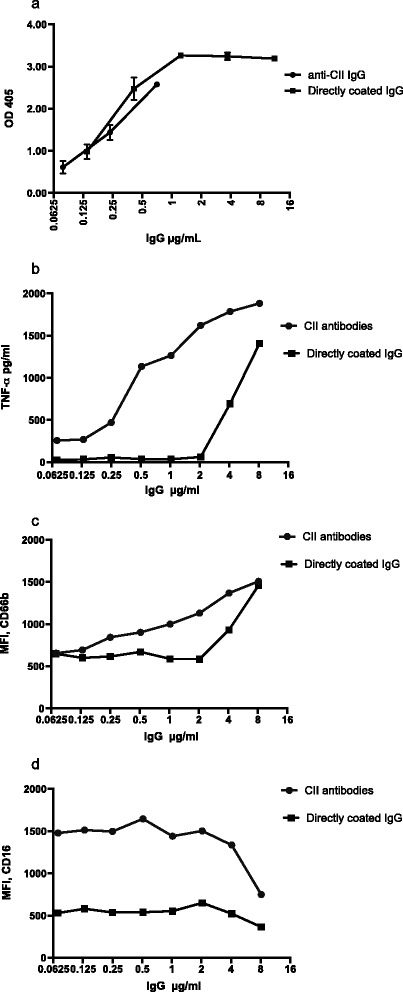
**Functional reactivity against IgG as part of surface-bound collagen type II autoantibody (anti-CII) immune complex and polyclonal IgG.** Amounts and functional reactivity against IgG as part of surface-bound collagen type II autoantibody (anti-CII) immune complex and polyclonal IgG directly coated to identical enzyme-linked immunosorbent assay (ELISA) plates. **(a)** ELISA reactivity of IgG against type II collagen (CII) aligned to known concentrations of directly coated IgG; these anti-CII levels (μg/ml) were thereafter used in the comparisons below. **(b)** Peripheral blood mononuclear cell-derived tumor necrosis factor alpha (ΤΝFα). **(c)**, **(d)** Polymorphonuclear granulocyte expression of CD66b and of CD16 respectively were compared for different concentrations of anti-CII and polyclonal IgG. Panels depict one representative result out of two to four experiments performed for each comparison. MFI, mean fluorescence intensity; OD, optical density.

When the concentrations of anti-CII IgG in the anti-CII IC were aligned to known concentrations of human IgG bound to identical ELISA plates, we found a similar pattern of slope for both systems (Figure 2a). The concentration of anti-CII IgG was determined to be 8 μg/ml IgG anti-CII in the serum used in the experiments shown in Figure 1. This level corresponds to 14,740 AU anti-CII/ml in our previous publications [1-3]. From our earlier studies we know that ΤΝFα production was dependent on anti-CII concentrations [3]. We found that changes in PMN CD marker expression required a higher concentration of anti-CII than ΤΝFα responses from PBMC (Figure 2b vs*.* Figure 2c,d). When PBMC ΤΝFα responses against anti-CII IC were compared with directly coated IgG, there was a left shift in the slope of ΤΝFα response against anti-CII IC, implying that the responder cells needed lower amounts of anti-CII in the form of surface-bound IC than directly bound IgG to respond with comparable TNF production (Figure 2b). Similar left shifts were also observed for PMN expression of CD16 and especially CD66b (Figure 2c,d).

### Polymorphonuclear granulocyte responses to anti-CII immune complex correlate with anti-CII levels in RA patients

**Figure 3:**
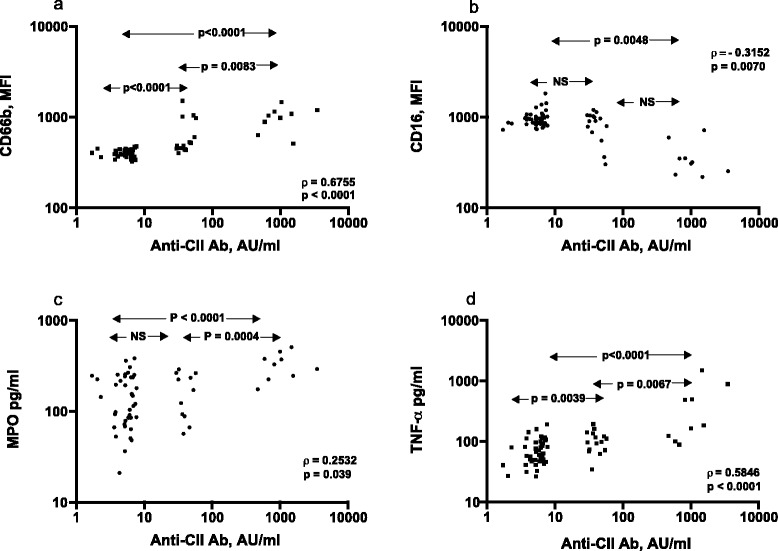
**Correlation between functional responses against collagen autoantibody immune complex and serum collagen autoantibody levels in 72 early rheumatoid arthritis patients. (a)**, **(b)** Data for polymorphonuclear granulocyte CD66b and CD16 expression. **(c)**, **(d)** Data for polymorphonuclear granulocyte supernatant levels of myeloperoxidase (MPO) and peripheral blood mononuclear cell supernatant levels of tumor necrosis factor alpha (ΤΝFα). Twenty-four of the patients have collagen type II autoantibody (anti-CII), nine of which represent the very high outlier group. The 48 anti-CII-negative patients chosen were those with the lowest anti-CII levels detectable with rheumatoid factor (*n* = 24) and without rheumatoid factor (*n* = 24) in the full rheumatoid arthritis cohort (*n* = 274). Statistics performed with the Spearman rank correlation test. AU, arbitrary units; MFI, mean fluorescence intensity; NS, nonsignificant.

Anti-CII IC-induced changes in PMN CD markers showed a strong correlation to anti-CII levels in the sera used to prepare the anti-CII IC. Expression of CD66b showed strong correlation (Spearman’s ρ = 0.675; *P* <0.0001), similar to PBMC ΤΝFα responses (ρ = 0.585; *P* <0.0001). Changes in PMN CD16 levels showed a negative correlation to anti-CII levels (ρ = –0.315; *P* <0.0001). Supernatant MPO levels showed a weaker correlation with anti-CII levels (ρ = 0.253, *P* <0.039; Figure 3). We found low supernatant IL-17 levels, without any correlation to anti-CII (data not shown). There were no differences in any functional measure between ICs prepared with sera from anti-CII-negative patients with and without RF (data not depicted).

### Polymorphonuclear granulocyte responses to anti-CII immune complex associate with acute phase reactants and radiological destruction during 2 years after RA diagnosis

In agreement with our earlier studies, baseline serum anti-CII levels and PBMC-derived ΤΝFα responses against anti-CII IC associated with baseline CRP and ESR, respectively (Table 2).

PMN cell surface levels of CD66b associated with baseline CRP, with a similar trend for CD16. Reductions in CD16 expression on PMN associated with Larsen score at baseline, 1 year, and 2 years, whereas elevated CD66b expression was associated with a higher destruction rate between all investigated time points. Serum anti-CII levels and ΤΝFα responses by PBMC did not show such associations with radiological destruction (Table 2).

## Discussion

In previous studies we have shown that anti-CII IC can stimulate PBMC monocytes *in vitro* to produce proinflammatory cytokines, and that these responses are associated with inflammatory measures and joint erosions at the time of early serum sampling. The *in vitro* function of these antibodies thus corresponds to the clinical phenotype in a time-dependent manner, arguing that this *in vitro* system mimics one tentative pathogenic mechanism *in vivo* in the subset of RA patients with elevated serum anti-CII levels at the time of diagnosis [1-3]. In the present study we have used an analogous model to study PMN reactivity towards anti-CII ICs, using the subset of patients representing the highest and lowest anti-CII levels in a larger previously published serological study [1]. Changes in PMN cell surface markers CD66b and CD16 associate with acute-onset inflammation and especially with joint destruction during the first 2 years after RA diagnosis. Notably, the association with joint destruction was stronger than for PBMC responses recorded as ΤΝFα production in response to parallel anti-CII ICs. In agreement with our previously published studies on the full cohort, anti-CII serum levels and PBMC-derived ΤΝFα associated with baseline CRP and ESR, whereas anti-cyclic citrullinated peptide version 2 (CCP2) and RF associated with an increased rate of cartilage destruction after 2 years, again arguing that the anti-CII-associated acute-onset RA phenotype differs from the bad long-term prognosis associated with the traditional serological markers RF and anti-CCP2 [1,35,36].

The CAIA mouse arthritis model has demonstrated that anti-CII can initiate acute polyarthritis associated with PMN activation leading to endothelial cell adhesion and recruitment of these PMN to inflamed tissue. The effect was diminished by antileukoproteinase, a PMN serine protease inhibitor, implying that PMN are primarily involved in the process [37,38]. CAIA is an acute-onset arthritis model, where joint symptoms appear during 8 to 12 days after booster lipopolysaccharide injection [39]. We believe that the anti-CII-associated acute-onset RA phenotype is the human counterpart to CAIA in mice, and that the anti-CII IC *in vitro* models we have used in this article and in previous papers explain (some of) the pathogenic mechanisms responsible for the characteristics of this RA phenotype.

When we normalized the IgG levels in anti-CII ICs to concentrations of human IgG directly coated to ELISA wells, we found that anti-CII ICs were more efficient in stimulating both PBMC and PMN (Figure 2). This was found in repeated experiments. One obvious hypothetical difference is the orientation of the Fc fragments where antibodies bound to coated antigens can be presumed to expose their Fc fragments upwards towards Fc receptors of added responder cells, whereas direct coating can result in stochastic orientation. The positioning of autoantibody epitopes within the native CII molecule might also affect the functional outcome, in analogy with an earlier report showing that a combination of four monoclonal anti-CII antibodies with closely positioned epitopes within the CII molecule is arthritogenic, whereas other antibody combinations are not [40]. The degree of IgG glycosylation is also tentatively important, as IgG lacking galactosyl residues had defects in binding to Fc gamma receptors [41-43]. Current studies on these discrepant findings are ongoing. The issue is not trivial because the CII-coated surface system had a weak autostimulatory response on PBMC ΤΝFα production (Figure 2b) and resulted in a higher baseline CD16 expression than directly coated IgG (Figure 2d). Addition of polymyxin B to the anti-CII IC did not change the difference between the two surfaces in stimulating ΤΝFα levels or expression of CD markers, arguing against a lipopolysaccharide-mediated effect. These differences between surfaces, however, do not influence the outcome of the present RA cohort study, where all clinical comparisons were made with anti-CII IC-induced responses.

Cell stimulatory responses *in vivo* are probably associated with the availability of anti-CII IC for cells in the synovial fluid. Investigations by Holmdahl and colleagues have shown that anti-CII can adhere to intact joint cartilage after intraperitoneal or intravenous injection [44,45], but these studies do not explain how such ICs will be in contact with responder cells in the synovial fluid. Studies from Jasin and colleagues indicate that binding of CII antibodies to an intact cartilage surface is dependent on neutrophil elastase-induced damage of the thin layer of small proteins covering the intact joint cartilage [46-48]. This disruption of the covering layer is transient, and is restored already after 10 days in a rabbit model [49], a time span in agreement with our previous findings that elevated anti-CII levels are associated with laboratory signs of inflammation at the time of RA diagnosis, but not 3 months later [1].

Although primarily associated with the Th17 T-cell subset, IL-17 can also be produced from innate immune cells, including PMN [17,26,27]. However, in this investigation we found very low levels of IL-17 induced by anti-CII IC-stimulated PMN, using an ELISA detecting IL-17A produced by PMN. MPO levels in the same supernatants, however, were high and correlated to anti-CII levels, although we could not show any association with the clinical phenotype as for the PMN CD markers.

Besides activating PMN in the joint space and synovial tissue, anti-CII containing ICs can also attract new PMN to enter the inflammatory area. The classical explanation is complement activation, as has been shown for anti-citrullinated protein/peptide antibody (ACPA) where low-avidity antibodies are associated both with strong complement activation and higher rate of joint destruction [50]. Besides creating chemotactic intermediates such as anaphylatoxin C3a and especially C5a, iC3b produced after IC-induced classical complement activation and covalently bound to surfaces close to ICs will bind PMN via complement receptors. Another explanation is the chemotactic action of PMN-attracting chemokines, notably IL-8/CXCL8. We have earlier shown that PBMC monocytes produce IL-8 in response to anti-CII IC [3], and these findings are currently being investigated further in the context of PMN.

In the present study, due to practical cell culture limitations, we only analyzed a fraction (*n* = 72) of patients in the previous study (*n* = 274). Anti-CII levels in RA patients show a discrete small outlier group of individuals with very high anti-CII levels that is clearly separated from the remaining patients (see Figure 1 in [2]), and the anti-CII-negative patients chosen were those with the lowest anti-CII levels in the cohort, so the graphs in Figure 3 show three seemingly separate groups of patients.

This paper extends our previous studies of collagen antibodies and their functional association with a clinical RA phenotype with acute onset, to also encompass a tentative pathogenetic role of PMN in this phenotype. Because the corresponding autoantigen is clearly joint specific, we believe that the strong temporal association between the occurrence of anti-CII, their functional roles *in vitro* in stimulating PBMC monocytes [3] and PMN, and the temporally associated clinical RA phenotype are rather strong arguments that anti-CII is associated with events having mechanistic similarities with the anti-CII-induced CAIA model in rodents. In the same cohort in which we showed this association [2,3], we previously have investigated the prognostic impact of anti-CCP2 [36]. In that study, median anti-CCP2 levels dropped to approximately 60% of baseline levels in the first year in the anti-CCP2-positive patients, and stayed low during the 5-year follow-up, at the same time as the anti-CCP2-positive patients showed progressively worse outcome, especially concerning swollen joint counts and Disease Activity Score in 28 joints. Other studies have also shown these temporally divergent patterns with decreasing ACPA levels in parallel to clinical worsening for ACPA-positive RA patients compared with ACPA-negative RA patients [51]. The fact that changes in ACPA levels lack association with the appearance of the ACPA-associated clinical RA phenotype, as is the case for anti-CII, should be considered in attempts to link ACPA functions *in vitro* to RA pathogenesis.

## Conclusions

We have investigated the role of PMN in the acute-onset RA phenotype associated with anti-CII. Our results imply that PMN responses against anti-CII ICs are more closely associated with early joint erosions than are PBMC cytokine responses. This implicates a prominent pathogenic role of PMN in the joint destruction seen at the time of diagnosis in patients with the acute-onset RA phenotype characterized by high levels of anti-CII.

## Abbreviations

ACPA: Anti-citrullinated protein/peptide antibody
anti-CII: Collagen type II autoantibody
AU: Arbitrary units
CAIA: Collagen antibody-induced arthritis
CCP2: Cyclic citrullinated peptide version 2
CD: Cluster of differentiation
CII: Collagen type II
CRP: C-reactive protein
ELISA: Enzyme-linked immunosorbent assay
ESR: Erythrocyte sedimentation rate
IC: Immune complex
IL: Interleukin
MPO: Myeloperoxidase
PBMC: Peripheral blood mononuclear cells
PMN: Polymorphonuclear granulocytes
RA: Rheumatoid arthritis
RF: Rheumatoid factor
TNFα: Tumor necrosis factor alpha
